# Falls occurrence is related to loss of vibration perception and functional reach in diabetes: a retrospective study

**DOI:** 10.1186/1758-5996-7-S1-A43

**Published:** 2015-11-11

**Authors:** Milene Elias Dalfolo, Isabel de Camargo Neves Sacco, Ulisses Tirolo Taddei, Maria do Socorro Morais Pereira Simões, Nathalie Ferrari, Fadlo Fraige Filho, Cristina Dallemole Sartor

**Affiliations:** 1 Universidade de Sao Paulo/Universidade Federal de Sao Paulo, São Paulo, Brazil

## Background

Falls incidence has a higher prevalence in diabetic patients, but there are few information about the contribution of the specific complications of polyneuropathy. The identification of those factors can increase efficacy of preventive and therapeutic actions to avoid the tragic consequences of falls episodes.

## Aims

Identify the occurrence of falls in the last 12 months in patients with Diabetes Mellitus (DM) and describe its association with diabetic polyneuropathy (DPN) signs and symptoms and with a functional task.

## Materials and methods

A cross-sectional, retrospective data analysis of 409 community dwelling diabetic patients was performed to describe, compare and verify possible associations between the variables. We followed the recommendations of international guidelines [1] and defined two main groups: non-fallers (NF, no episode of falls in the past 12 months) and at risk of falling (RF, at least 1 fall in the past 12 months). Patients voluntarily participated in a campaign for prevention and detection of diabetes, during one day of activity. The clinical assessment was composed by (a) questionnaire of Michigan Neuropathy Screening Instrument, (b) tactile and vibration perception [2,3], (c) functional reach test and (d) self-reported falls occurrence questionnaire in the past 12 months. Descriptive statistics, T tests and Chi-Square tests were used to identify differences between groups (α=5%).

## Results

**Figure 1:**
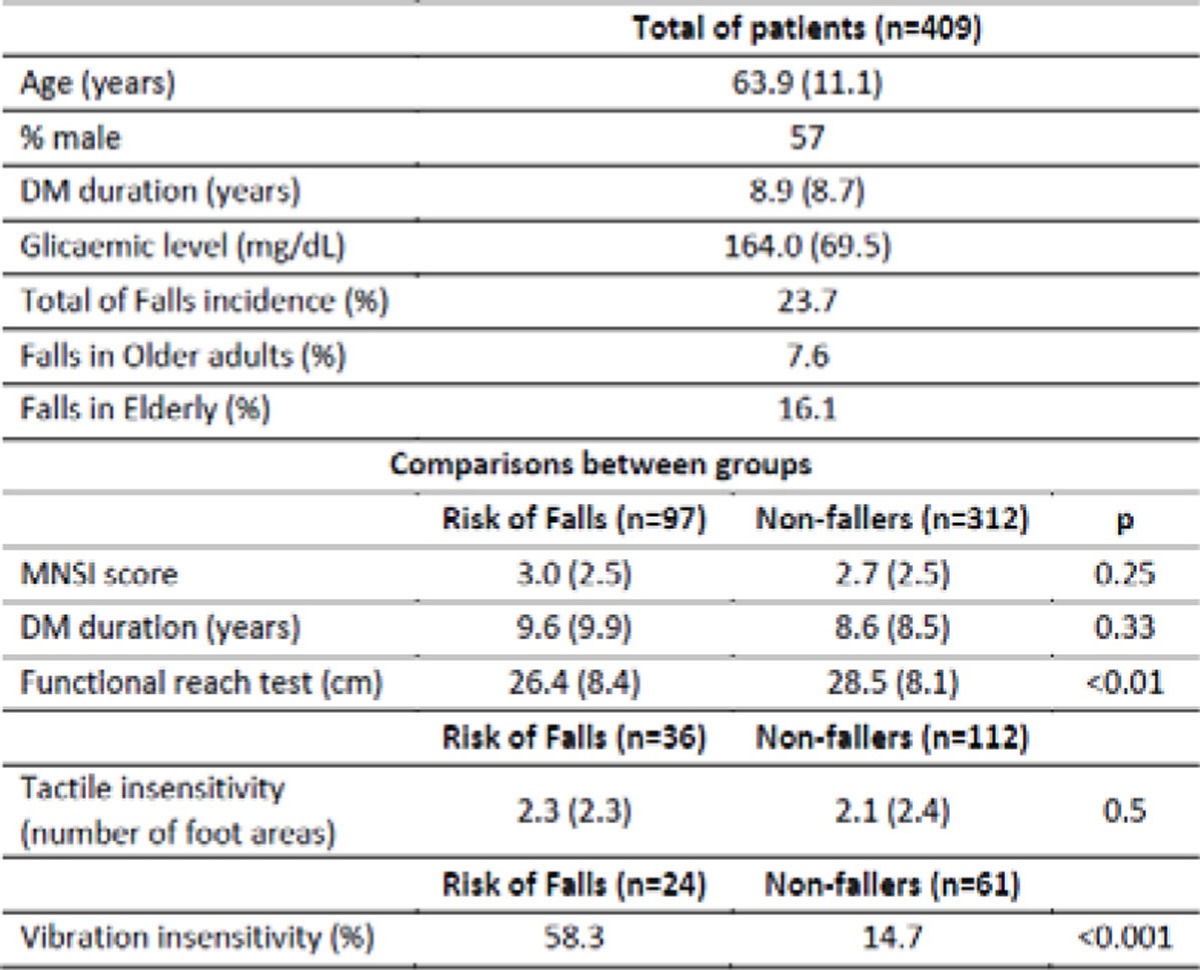
Description (mean and standard deviations) and comparisons between groups.

There were higher occurrences of falls in elderly DM patients than in older adults (Figure 1). There were no differences of DPN-related symptoms, time of DM onset and the number of tactile insensate foot regions between groups. However, the fallers presented decreased vibration perception and lower functional reach test score compared to non-fallers.

## Conclusion

DM individuals with decreased vibration perception and decreased functional reach test are exposed to higher risk of falls. Those clinical variables can be used to implement falls prevention programs for this population.

